# Postoperative Outcome of Combined Phacovitrectomy in Eyes with Excessive Myopia (>-30 D)

**DOI:** 10.1155/2023/7367922

**Published:** 2023-11-14

**Authors:** Hua Fan, Mingming Zhang, Radouil Tzekov, Zhuyun Qian, Jiasong Yang, XiaoLin Xie, Wensheng Li

**Affiliations:** ^1^Department of Retina, Shanghai Aier Eye Hospital, Shanghai, China; ^2^Department of Retina, Shanxi Aier Eye Hospital, Shanghai, China; ^3^Department of Cataract & Retina, Yancheng Aier Eye Hospital, Yancheng, China; ^4^Department of Ophthalmology, University of South Florida, Tampa, Florida, USA; ^5^Aier School of Ophthalmology, Central South University, Changsha, China; ^6^Shanghai Aier Eye Institute, Shanghai, China

## Abstract

**Background:**

To report the outcomes of phacoemulsification combined with vitrectomy in eyes with extreme myopia (-30 diopters or more). *Case Presentation*. Three patients with cataract, vitreous opacities, and extreme myopia of more than -30 diopters underwent a combined surgical procedure of cataract extraction combined with vitrectomy. Postoperative refractive correction of the three cases ranged from -1.0 D to -2.5 D spherical equivalent. There was an obvious hyperopic shift of all cases. All patients noted a significant improvement in uncorrected and best-corrected visual acuity from 0.4 to 0.8 in case 1, from CF/70 cm to 1.0 in case 2, and from 0.12 to 0.5 in the right eye and 0.15 to 0.2 in the left eye in case 3. Vitreous floaters disappeared in all cases. No complications were noted during follow-up.

**Conclusions:**

To the best of the authors' knowledge, these represent the first reported clinical cases of combined cataract extraction+vitrectomy surgery in eyes with extreme (>-30 D) myopia. Our results support the notion that phacoemulsification combined with vitrectomy may be a good therapeutic option for cataracts and vitreous floaters in cases with extreme myopia.

## 1. Background

With the elongation of eyeball, highly myopic eyes show distinctive ocular structural changes, such as thinning of the choroid and retina and posterior scleral staphyloma, which affect outcomes of ocular surgery and bring difficulty and risks during surgery. Extreme long axial length was also reported associated with unpredictable refractive result [1]. Cases of high myopia with a spherical equivalent of more than -30 D are rare. In this case report, we presented surgical outcomes of cases with a spherical equivalent of more than -30 D.

## 2. Case Presentation

### 2.1. Case 1

A 41-year-old woman, with a history of high myopia, was presented to our eye clinic with decreased visual acuity in both eyes for 6 months. The uncorrected visual acuity (UCVA) was 0.02 and best-corrected visual acuity (BCVA) was 0.4 with an axial length of 35.6 mm (spherical equivalent more than -30.0 diopters (D)) in her right eye and BCVA 0.02 with an axial length of 33.3 mm (spherical equivalent −25.0 diopters (D)) in the left eye. There was a localized retinal detachment in her left eye and cataract with vitreous opacities in both eyes. Optical coherence tomography (OCT) showed a macular hole in her left eye with a foveal retinal detachment. A diagnosis of extreme high myopia with vitreous opacity in both eyes, cataract in both eyes, and retinal detachment with macular hole in the left eye was established. Combined surgical procedure including vitrectomy combined with phacoemulsification and intraocular lens (MA60MA IOL Alcon, -5 D) implantation was performed in the right eye, and a combined surgical procedure including vitrectomy combined with phacoemulsification, internal retinal membrane peeling, and silicon oil tamponade was performed in the left eye. At one week after the surgery, UCVA and BCVA in the right eye improved to 0.4 and 0.5 (−1.00 D sph/−4.00 D cyl × 65°), and retinal reattachment was observed with increased BCVA of 0.1 in the left eye. At 6 months after the surgery, the BCVA of her right eye improved to 0.8, and BCVA of the left eye was stable after silicon oil removal and combined IOL implantation. No adverse effects were observed during the course of treatment and follow-up.

### 2.2. Case 2

A 38-year-old man complained of decreased visual acuity and floaters in his right eye within the past 2 years. UCVA was CF/30 cm and BCVA of the right eye was CF/70 cm with a spherical equivalent of more than -30 D and axial length of 37.7 mm. His left eye had no light perception. Nuclear cataract related to high myopia and lens subluxation was observed in the right eye. Ultrasound B-scan examination showed posterior vitreous detachment and vitreous opacity in the right eye. A diagnosis of extreme high myopia, cataract, and lens subluxation in the right eye was established. A combined surgical procedure including phacoemulsification and intraocular lens implantation (MA60MA, -3 D) plus vitrectomy was performed in the right eye. Due to the history of lens subluxation and as a preventive measure against recurrence, a capsular tension ring was implanted in the bag during surgery. After surgery, BCVA was improved to 0.4. During 10-month follow-up, visual acuity was stable without any further complications.

### 2.3. Case 3

A 52-year-old man complained of gradually decreased visual acuity and floaters in both eyes during the past 20 years. UCVA was CF/10 cm in both eyes. BCVA was 0.15 (right eye) and 0.12 (left eye) with more than -30 D spherical equivalent in both eyes. The axial length of his right eye is 33.3 mm and in the left eye was 32.6 mm. Moderate nuclear cataract was observed in both eyes. Ultrasound B-scan examination showed moderate vitreous opacities in both eyes. A diagnosis of extreme high myopia, cataract, and vitreous opacity in both eyes was established. Vitrectomy surgery combined with phacoemulsification (assisted by femtosecond laser) and intraocular lens implantation (MA60MA, -1 D in the right eye and MA60MA, 1 D in the left eye) was performed in both eyes. After surgery, his visual acuity improved in both eyes (BCVA 0.5 with SE of -1 D in the right eye and BCVA 0.2 with SE of -1.5 D in the left eye). During 2-year follow-up, his visual acuity was stable in both eyes without any further complications.

## 3. Discussion

In our cases of more than -30 D high myopic eyes, axial length ranged from 32.6 mm to 37.5 mm (Table 1). All cases described here presented with nuclear cataracts and vitreous floaters. One case presented with lens subluxation. It is reported that there was a statistically significant association between high myopia and incident of nuclear cataract [2]. High myopia was also related to posterior vitreous detachment and vitreous opacities [3]. Since in all three cases the chief complaint was decreased vision and visual disturbance due to floaters, a combined surgical procedure including phacoemulsification with vitrectomy was chosen as a therapeutic approach. Postoperatively, besides improved visual acuity (both uncorrected and best-corrected), visual disturbance due to floaters disappeared, and all patients were satisfied with the outcome.

Vitreous and cataract surgery was reported to be more challenging in eyes with high myopia [4, 5]. In such cases, the sclera and choroid appear to be thinner than usual and zonules are loose [6]. Based on the history of lens subluxation and to prevent a recurrence, capsular tension ring was implanted in case 2. In case 3, femtosecond laser-assisted cataract surgery was performed in order to protect the zonule [7]. Good postoperative outcomes following phacoemulsification combined with vitrectomy were observed in cases with myopia with cataract and vitreous opacities. In 2013, our team reported a prospective study of refractive lens exchange (RLE) by phacoemulsification with posterior chamber intraocular lens (IOL) implantation combined with simultaneous pars plana vitrectomy (PPV) in the management of high myopia [8]. In that study, 45 eyes of 26 patients with preoperative myopia greater than –12.5 D were included, and the average postoperative BCVA improved dramatically after surgery. Furthermore, 82.2% of the eyes had a BCVA of 0.5 or better during the four-year follow-up [8]. However, risks of postoperative retinal tears and rhegmatogenous retinal detachment were related to high myopia. During vitrectomy and postoperatively, preventative laser photocoagulation treatment should be considered where necessary.

Extreme axial length was also reported to be associated with unpredictable refractive result. It was reported that the hyperopic shift and biometry prediction error with lower-power IOLs were common [1, 5]. Considering hyperopic shift and patients' request of better near vision postoperatively to maintain a near-sighted lifestyle, the rationale of choice of the target spherical equivalence (TSE) was determined by the reading distance (RD) using the formula TSE = −1/RD (m). Intraocular lens power was determined by the SRK/T formula in all cases. It has been reported that increasing axial length and lower-power IOLs lead to hyperopic shift postoperatively [9]. Compared to normal eyes and mildly myopic eyes, highly myopic eyes are more prone to refractive surprise. The reported hyperopic shift ranged from 1 to 4 D [10, 11]. In our cases, the average hyperopic shift ranged from 2.0 to 1.41 D (Table 1). The biometry prediction error may be related to the extreme axial length and the inaccuracy of measurement. Capsular tension rings (CTRs) are implanted during cataract surgery to stabilize the position of the capsular bag and keep the IOL centered after surgery in eyes. In patients with axial myopic astigmatism, CTR can effectively increase the rotational stability of a toric IOL [12]. However, it was also reported that implantation of a CTR had no consistent effect on refractive outcomes compared with routine phacoemulsification in highly myopic eyes [13]. In our cases, capsular tension ring was implanted in case 2 for lens subluxation. It seems less hyperopic error in case 2 than case 1 and case 3; however, there were only 3 cases presented, and a further study is needed to reach a conclusion.

Our cases confirm that a satisfactory outcome can be achieved in phacoemulsification combined with vitrectomy for extreme high myopia (more than -30 D), cases that are rare in the general population.

## Figures and Tables

**Table 1:** Preoperative and postoperative findings.

Parameter	Case 1 (OD)	Case 2 (OD)	Case 3 (OD)	Case 3 (OS)
Axial length (mm)	35.57	37.72	33.28	32.64
ACD (mm)	3.61	3.23	2.96	2.92
Preoperative CDVA	0.4	CF/70 cm	CF/10 cm	CF/10 cm
Postoperative CDVA	0.8	0.4	0.5	0.2
Target SE (D)	-3.96	-4.41	-3.50	-2.50
Postoperative SE (D)	-2.00	-3.00	-1.50	-1.00
Biometry prediction error (D)	1.96	1.41	2	1.50

SE: spherical equivalent; ACD: anterior chamber depth; CDVA: corrected distance visual acuity; LP: light perception.

## Data Availability

All data generated or analysed during this study are included in this published article.
